# Tailoring the Fire-Retardant Efficacy of Intumescent Coatings via Optimal Selection of Epoxy Binder Molecular Weight

**DOI:** 10.3390/polym18182201

**Published:** 2026-09-09

**Authors:** Daria Kuznetsova, Nikolay Yashin, Vyacheslav Subbotin, Victor Avdeev

**Affiliations:** Faculty of Chemistry, Lomonosov Moscow State University, Moscow 119991, Russia; mallolo1111@gmail.com (D.K.); subbotin.v@ograx.ru (V.S.); avdeev@highp.chem.msu.ru (V.A.)

**Keywords:** epoxy resin, intumescent coating, molecular weight, fire-retardant efficacy, expandable graphite, thermal degradation

## Abstract

Epoxy resins are widely used as binders in intumescent fire-retardant coatings; however, the effect of their molecular weight on the fire-retardant performance of coatings with different intumescent mechanisms remains insufficiently understood. In this study, five commercial bisphenol A-based epoxy resins with number-average molecular weights (*M_n_*) ranging from 375 to 1520 g/mol were characterized by HPLC, NMR, DSC, and TGA, and subsequently formulated into intumescent coatings of two types: classical ammonium polyphosphate/pentaerythritol/melamine (APP/PER/MEL) systems and expandable graphite (EG)-based systems. The fire-retardant efficacy and expansion factor were evaluated using a standard fire test and thermal shock exposure. In the APP/PER/MEL coatings, the optimal fire-retardant efficacy of 55 min was achieved at an intermediate *Mn* (645 g/mol) and an optimal pore structure (mean equivalent pore diameter *d*_eq_ = 41 μm), whereas both low- and high-molecular-weight resins led to distinct structural defects—macrocavities and through-thickness cracks, respectively, as revealed by SEM. In the EG-based coatings, a monotonic decrease in both the expansion factor (from ~2000% to ~1000%) and fire-retardant efficacy (from 65 to 52 min) was observed with increasing *M_n_*, with the bisphenol A-based epoxy resin having a molecular weight of 375 g/mol providing the best performance. The observed differences were attributed to the effect of melt viscosity on char expansion and structural integrity. These findings demonstrate that the molecular-weight range of the epoxy binder can be selected according to the expansion capacity of the intumescent system, providing a practical guideline for the formulation of high-performance epoxy-based fire-protective coatings.

## 1. Introduction

Fire protection of load-bearing steel structures is essential for maintaining their structural integrity under high-temperature exposure, particularly in the oil and gas industry, where hydrocarbon fires can develop rapidly and reach severe thermal conditions [1,2,3,4,5,6,7]. Thin-film intumescent coatings are widely used as passive fire protection systems for this purpose. Upon heating, these coatings undergo a sequence of physicochemical transformations that result in the formation of an expanded char layer, which acts as a thermal barrier and delays the heating of the protected steel substrate [8,9,10].

The effectiveness and durability of intumescent coatings depend strongly on the properties of the polymer binder, which serves not only as a film-forming component but also plays a decisive role in char formation, melt rheology, and adhesion to the substrate [11,12,13,14,15,16,17,18,19]. Among the various polymer classes investigated for intumescent coatings, epoxy binders are particularly attractive for the protection of steel structures because of their high adhesion and resistance to aggressive environments [13,14,20,21,22]. However, the properties of an epoxy binder are determined not only by its chemical composition but also by its molecular-weight characteristics and curing chemistry. In contrast to linear thermoplastics, epoxy systems form a three-dimensional crosslinked network upon curing, and the structure and functionality of the curing agent can therefore substantially affect the thermal degradation, rheological behavior, and char-forming characteristics of the coating [23].

A previous systematic study of polyvinyl acetate (PVA) binders demonstrated that molecular weight can have a pronounced and nonlinear effect on the fire-retardant performance of an intumescent coating based on ammonium polyphosphate (APP), pentaerythritol (PER), and melamine (MEL). Low-molecular-weight PVA resulted in rapid but unstable foaming, whereas excessively high-molecular-weight PVA produced a rigid char prone to cracking; the optimum performance was obtained within an intermediate molecular-weight range [24]. These results indicate that optimization of binder molecular weight within a given polymer class can be an effective approach to simultaneously improving the fire-retardant and mechanical characteristics of intumescent coatings. However, PVA-based fire-protective formulations are primarily suitable for interior applications because of their limited resistance to prolonged atmospheric exposure.

To date, this approach has not been systematically extended to epoxy binders, which would facilitate the development of weather-resistant fire-protective coatings. Only limited studies have addressed the effect of epoxy oligomer molecular weight on intumescent behavior; for example, Wang and Yang [25] reported an influence of molecular weight on melt viscosity and char porosity in a single waterborne formulation. Moreover, the structural characteristics of epoxy binders, including the molecular weight of the epoxy oligomer has not been systematically investigated in conventional APP/PER/MEL systems. An even less explored area is the use of oxidized/expandable graphite, despite the fundamentally different expansion mechanism of graphite-based intumescent systems and the strong dependence of their performance on the rheological characteristics of the polymer matrix [16,17,18,21,26].

Accordingly, the specific research gap addressed in this work concerns the lack of systematic knowledge of how epoxy binder molecular weight affects the performance of intumescent coatings containing fundamentally different intumescent systems. The present study therefore investigates a bisphenol A-based epoxy oligomer with systematically varied molecular weight in two model formulations: the conventional APP/PER/MEL system and a system based on oxidized/expandable graphite.

This comparative approach makes it possible to distinguish the effects of epoxy binder structure from those arising from the nature of the intumescent component and to identify the binder characteristics that provide the most favorable combination of fire-retardant performance, char integrity, and mechanical stability. Ultimately, the results provide a basis for the rational development of weather-resistant fire-protective coatings for steel structures intended for long-term service under diverse climatic and geographical conditions.

## 2. Materials and Methods

### 2.1. Materials

Epoxy resins based on bisphenol A with different number average molecular weight M_n_ were supplied by Nan Ya Plastics Corporation, Kaohsiung City, Taiwan (Table 1). Triethylenetetramine (TETA, Huntsman Corp., Conroe, TX, USA) was used as a curing agent. The intumescent system of the compositions include ammonium polyphosphate KYLIN APP201 (Shifang Changfeng Chemical Co., Deyang, China, crystalline phase II, avg. degree of polymerization ≥ 50), zinc borate HT-207 (Jinan Taixing Fine Chemicals Co., Jinan, China, weight fraction B_2_O_3_ 45–80%, ZnO 36–39%, loss on ignition at 450 °C was 13–15%), melamine (Sichuan Golden-Elephant Sincerity Chemical Co., Meishan, China, purity ≥ 99.8%), expandable graphite EG-250-80 (Qingdao Freyr Graphite Co., Pingdu, China, carbon content 96.1%); titanium dioxide TiOx-280 (CRIMEA TITAN, weight fraction of TiO_2_ ≥ 93.5%, rutile form ≥ 98%), pentaerythritol (Metafrax Chemicals, Perm, Russian, purity ≥ 95%, moisture and volatile content ≤ 0.2%), magnesium silicate fiber (Orenburg Minerals JSC, Yasny, Russia, weight fraction SiO_2_ 45%, MgO 40%, Fe_2_O_3_ 1.5%); antimony (III) oxide (Hsikwangshan twinkling star Co., Lengshuijiang, China, Sb_2_O_3_ content 99.84%). Xylene (AMK-khimiko JSC, Moscow, Russia, petroleum grade A) was used as solvent.

### 2.2. Intumescent Fire-Retardant Formulations

The effect of the binder molecular weight on the fire-retardant performance of the coatings was investigated using two model intumescent compositions: one based on ammonium polyphosphate, melamine, and pentaerythritol (Table 2), and the other based on expandable graphite (Table 3).

Before the preparation of the fire-retardant paint formulations, the solid epoxy resins were dissolved in xylene to obtain 80 wt.% solutions. The dissolution was carried out at 80 °C under stirring for 1 h. The solid content of the solutions was determined by ISO 3251:2019.

The fire-retardant formulations were produced using a laboratory dissolver (Biuged Instruments, Guangzhou, China, model BGD 750/1). A calculated amount of solvent and functional additives (plasticizer, dispersion agent and rheological modifier) was added to the epoxy resins. The components of the IS were then introduced gradually under continuous stirring at 1500 rpm. After all filler components were incorporated, the resulting compositions were homogenized for an additional 5 min at 2000 rpm.

Prior to fire-retardant coating application, the prepared compositions were blended with TETA hardener in a stoichiometric amount calculated based on the epoxy equivalent weight of the resin (Table 1).

### 2.3. Characterization

#### 2.3.1. Methods for the Characterization of Epoxy Resins

The oligomeric composition of the epoxy resins was analyzed by reversed-phase high-performance liquid chromatography (RP-HPLC) using an Agilent 1260 Infinity II system (Agilent Technologies, Santa Clara, CA, USA) equipped with a diode-array detector. Separation was performed on an Agilent Poroshell 120 EC-C18 column (particle size 2.7 µm, 4.6 × 150 mm) maintained at 40 °C. The mobile phase consisted of acetonitrile and deionized water. The eluent flow rate was 1.0 mL/min, and the injection volume was 3.0 µL. Detection was carried out at a wavelength of 224 nm. Dibutyl phthalate (DBP) was added to each sample as an internal standard to monitor the quantitative composition and ensure injection reproducibility.

^1^H NMR spectra (Figures S1–S5) of the epoxy resins were acquired on a Varian VNMRS 400 MHz spectrometer (Agilent Technologies, Santa Clara, CA, USA) at 30 °C in CDCl_3_, with chemical shifts referenced to the residual solvent signal (δ = 7.26 ppm).

The polymer matrices cured with TETA were analyzed by differential scanning calorimetry (DSC) in accordance with ISO 11357-2:2020 using a heat-flux differential scanning calorimeter (DSC Q20, TA Instruments, New Castle, DE, USA). Samples were sealed in aluminum pans and measured under a high-purity nitrogen atmosphere (≥99.999%) at a flow rate of 100 mL/min. The temperature program ranged from −50 to 250 °C at a heating rate of 10 °C/min to accurately determine the glass transition temperature (*T_g_*).

#### 2.3.2. Methods for Assessing Fire-Retardant Efficacy

The tests were performed on steel plates measuring 250 × 250 × 5 mm, equipped with a thermocouple hole drilled on the unexposed side, which was thermally insulated. Each formulation was tested in duplicate to ensure reproducibility of the results. The compositions were applied layer by layer using a film applicator until a total dry film thickness of 5 mm was achieved. After each layer, the coatings were conditioned for 24 h at room temperature and 1 h at 80 °C. Prior to testing, the finished coatings were conditioned for at least 168 h at 23 ± 2 °C and 50 ± 5% relative humidity. The dry film thickness was measured with a magnetic thickness gauge.

The fire-retardant efficacy of intumescent coatings was determined on a stand for conducting fire tests of small-sized steel structures in the standard (“cellulose”) fire mode according to British Standard BS 476-20:1987, which is described by the following temperature–time relationship:

(1) T=345 log108t+1+20,

where T is the temperature in the furnace at time *t* (°C) and *t* is the time counted from the start of the test (min).

The fire-retardant efficacy was quantified as the time required for the temperature on the unexposed (back) surface of the coated steel plate to reach 500 °C, measured from the onset of heating with an accuracy of ±1 min.

#### 2.3.3. Measurement of the Expansion Factor

Thermal expansion under rapid heating (thermal shock) was evaluated as the arithmetic mean of three replicate specimens of the same formulation. Square specimens with dimensions of 40 × 40 × 1 mm were cut from the cured coatings. The initial dry film thickness *h*_0_ was measured using a magnetic thickness gauge. The specimens were then placed on a non-combustible, heat-resistant substrate and exposed in a muffle furnace at 600 °C for 5 min. After cooling to ambient temperature, the thickness of the expanded char layer *h*_1_ was measured using a caliper. The expansion factor *K* (%) was calculated using the formula:

(2) $$K=\left(h_{1}-h_{0}\right)/h_{0}\cdot 100,$$

#### 2.3.4. Thermal Analysis

Thermogravimetric analysis (TGA) of the cured epoxy resins and the corresponding coatings was performed using a thermal analyzer (STA 449, NETZSCH-Gerätebau GmbH, Selb, Germany). Measurements were carried out in corundum crucibles under an air atmosphere at a flow rate of 100 mL/min. The samples were heated from 70 to 900 °C at a heating rate of 10 °C/min.

#### 2.3.5. Characterization of Chair Layer

SEM observations were performed using a Vega 3 microscope (Tescan, Brno–Kohoutovice, Czech Republic) equipped with secondary and backscattered electron detectors and operated at an accelerating voltage of 20 kV. Quantitative analysis of the pore structure was performed using Fiji/ImageJ software (ver. 2.18.0). The SEM images were segmented to distinguish pores from the carbon matrix, and the projected area of each individual pore was measured. The equivalent pore diameter *d_eq_* was calculated from the measured pore area *A* according to $d_{eq}=\sqrt{A/\pi }$. Pore-size distributions were expressed as the number fraction of pores within predefined diameter intervals.

Fourier transform infrared (FTIR) spectra were recorded using a Tensor 27 spectrometer (Bruker Optik GmbH, Baden-Württemberg, Germany).

## 3. Results and Discussion

### 3.1. Characterization of Epoxy Resins

**Figure 1:**
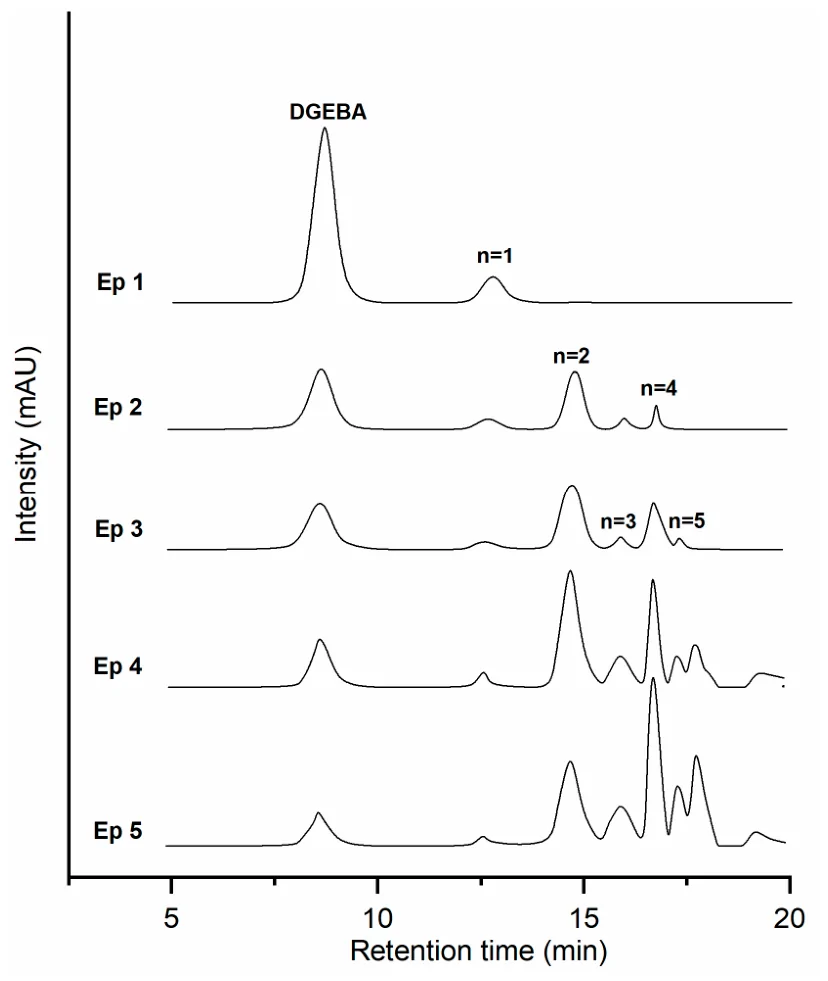
RP-HPLC chromatograms of the epoxy resins.

**Figure 2:**
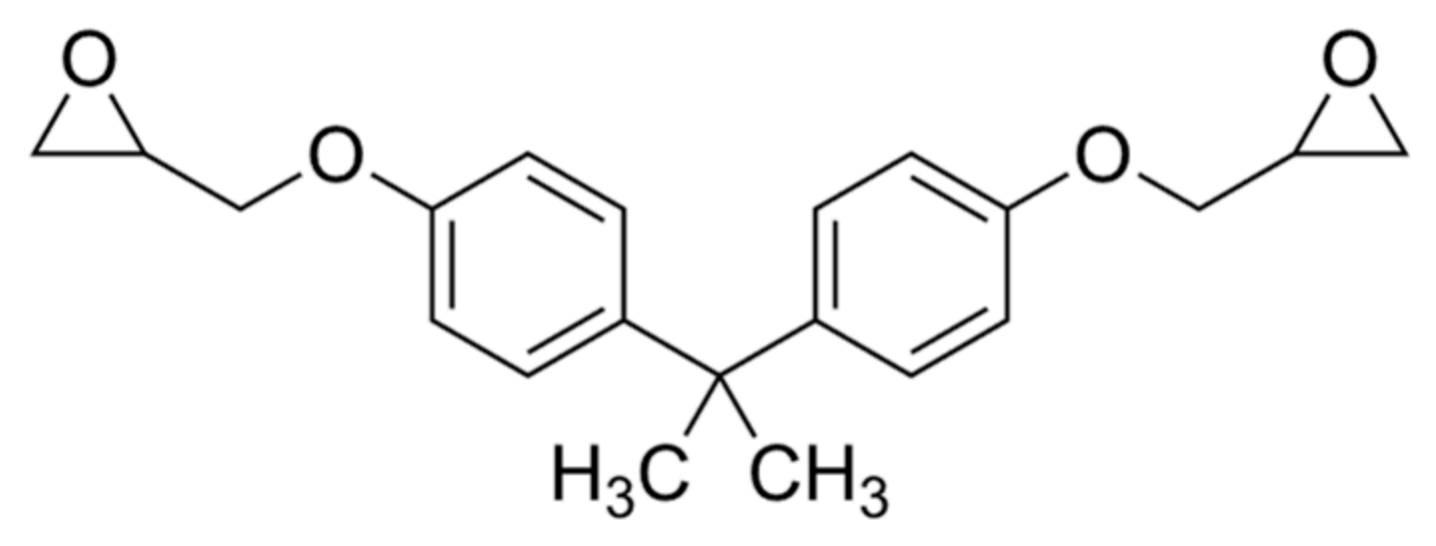
Chemical structures of DGEBA.

The oligomeric composition of five industrial epoxy resins was investigated using RP-HPLC. A homologous series of bisphenol A-based epoxy oligomers was identified in the chromatograms of all samples (Figure 1). As M_n_ increases (from Ep1 to Ep5), a progressive decrease in diglycidyl ether of bisphenol A (DGEBA, Figure 2) content is observed. The retention time of the oligomeric fractions increases regularly with the degree of polymerization (n), which is attributed to the increase in molecular hydrophobicity associated with chain extension. The applied RP-HPLC method, using a Poroshell 120 EC-C18 column with an acetonitrile-based mobile phase, ensures effective separation of low- and medium-molecular-weight oligomeric homologs. However, owing to the high hydrophobicity of long-chain epoxy oligomers (*n* ≥ 5), their elution on the C18 column is either significantly delayed or incomplete, with partial irreversible adsorption onto the stationary phase [27]. Consequently, the quantitative determination of higher-molecular-weight fractions (*n* > 5) is limited within the scope of the present methodology.

Analysis of the oligomeric distribution revealed a clear classification of the resins into three distinct groups based on their predominant fractions and molecular weights (Table 4). The first group comprises the low-molecular-weight resin Ep1, characterized by a DGEBA content exceeding 80%. The second group includes Ep2 and Ep3, which exhibit a broader molecular weight distribution with a notable contribution of oligomers ranging from trimers (*n* = 2) to pentamers (*n* = 4). The third group consists of the high-molecular-weight solid resins Ep4 and Ep5, in which oligomeric species with a degree of polymerization *n* ≥ 2 predominate.

Additionally, ^1^H NMR spectroscopy was used to estimate the average degree of oligomerization of resins Ep1–Ep5 according to the method proposed in [28], which is based on comparing the intensity of the signal of the methine or one of the methylene protons of the epoxy ring with the intensity of one of the signals of the aromatic protons. The signals selected for this analysis should be well resolved and free from overlapping with other signals. In the present study, it was used the ratio of the signal intensity of the epoxy-ring methine proton at δ_H_ = 3.35–3.40 ppm to that of the methine protons of the aromatic fragments at δ_H_ = 6.85–6.90 ppm, based on the ^1^H NMR spectra of resins Ep1–Ep5 shown in Figures S1–S5. The resulting ratios were then compared with the calculated theoretical values for DGEBA oligomers, allowing the average degree of oligomerization to be determined. The average degree of oligomerization increased by approximately one unit across the Ep1–Ep5 resin series (Table 5). These data are generally in good agreement with the results obtained by RP-HPLC.

**Figure 3:**
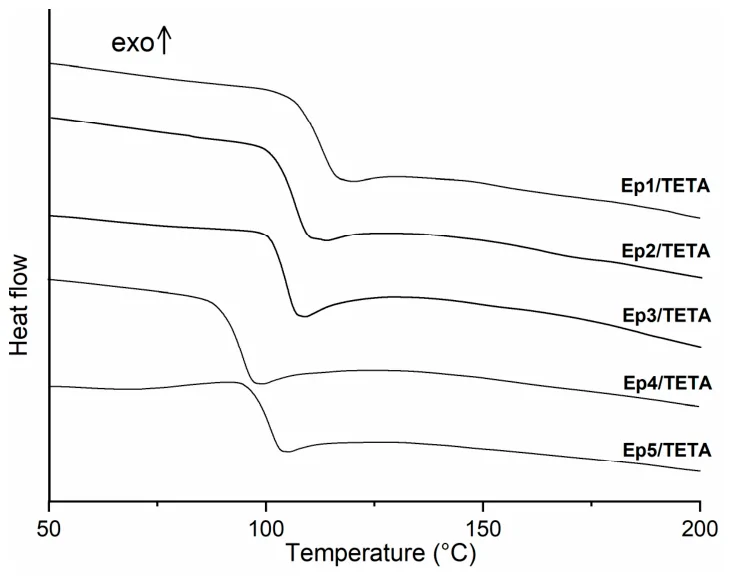
DSC curves of the cured epoxy resins under N_2_ atmosphere.

The thermal behavior of the investigated epoxy resins cured with TETA was studied by DSC. The heating curves of all samples (Ep1/TETA–Ep5/TETA) exhibited a distinct step-like transition corresponding to the glass transition of the crosslinked polymer (Figure 3). The DSC analysis revealed that T_g_ decreases with increasing M_n_ within the series of resins, which can be attributed to the decreasing rigidity of the polymer matrix and the increasing segmental mobility [29,30]. The highest T_g_ value was recorded for Ep1/TETA (113 °C), which corresponds to the most densely crosslinked network formed by low-molecular-weight DGEBA. The lowest T_g_ value was observed for Ep4/TETA (Table 6).

The difference in glass transition temperatures between the two solid resins (Ep4 and Ep5) arises from differences in their oligomeric composition. According to the HPLC data (Table 4), Ep4 contains the highest proportion of higher oligomers, which introduce steric hindrance and restrict the mobility of reactive sites. Furthermore, compared with Ep5, a greater amount of non-eluting material remained on the column for Ep4, which may indicate the presence of not only a high-molecular-weight tail but also additional hydrophobic components. After the curing reaction, these components may remain in the polymer matrix as unreacted fragments and act as an internal plasticizer.

**Figure 4:**
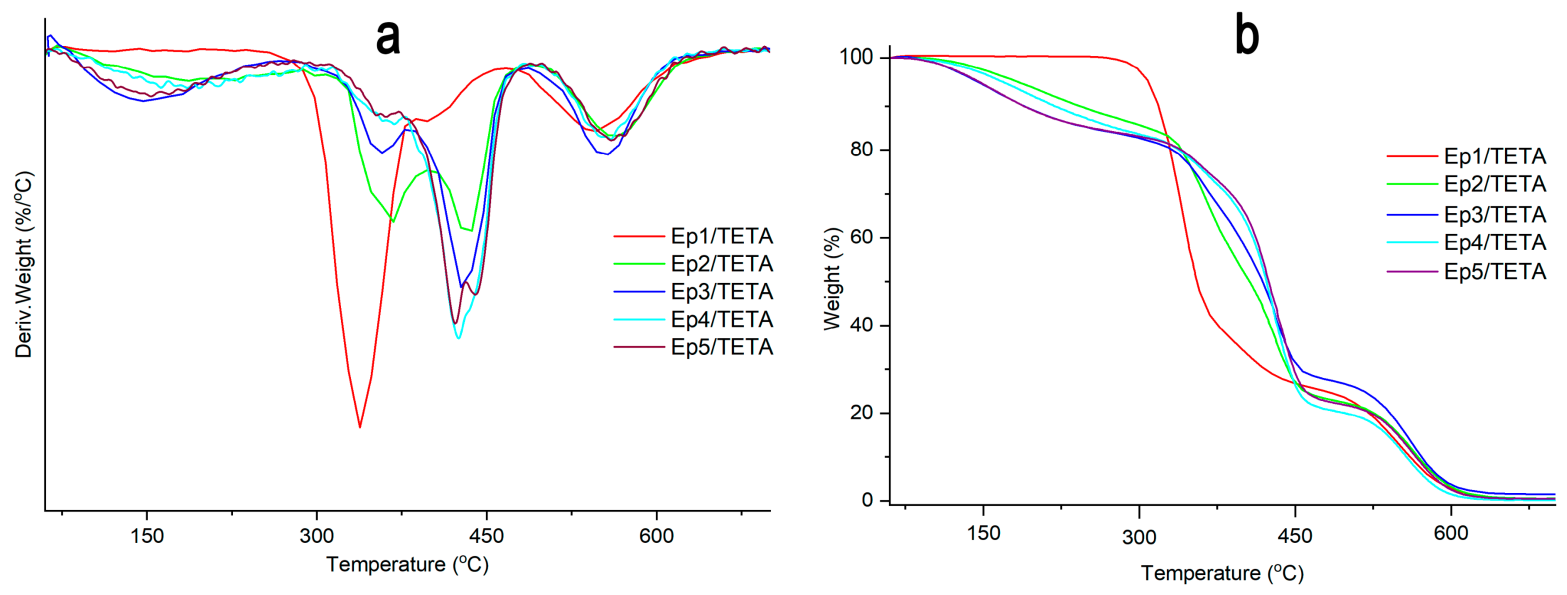
DTG (**a**) and TG-curves (**b**) of the cured epoxy resins under O_2_ atmosphere.

The TGA results for the cured epoxy resins are presented in Figure 4 and Table 7. The thermo-oxidative degradation process of all the investigated specimens exhibited a multistage character, as evidenced by the presence of several distinct maxima in the mass loss rate on the derivative thermogravimetric (DTG) curves. For all samples, the stage of active decomposition of the polymer matrix, characterized by the greatest mass loss, was observed in the 300–450 °C range. With an increase in M_n_ of the resins, a decrease in the onset temperature of 5% mass loss (T_5%_) was observed, along with notable changes in the DTG profiles of the studied specimens. Ep1/TETA remained thermally stable up to 300 °C, and its main degradation stage occurred within a relatively narrow temperature interval, featuring a pronounced DTG peak with a maximum mass loss rate temperature (T_max1_) of 340 °C Upon transitioning to the higher-molecular-weight samples, the main degradation stage shifted toward higher temperatures, and a new DTG peak with its own temperature maximum (T_max2_) emerged, while the mass loss rate at T_max1_ decreased. At temperatures above 500 °C, the combustion of the carbonaceous residue began, culminating in the complete decomposition of all samples at approximately 600 °C.

The observed differences in the thermal behavior of the samples can be attributed to the increasing structural heterogeneity of the polymer network with rising molecular weight distribution of the resins. The thermal degradation of amine-cured epoxies is strongly influenced by the concentration and nucleophilicity of the nitrogen atoms within the crosslinked structure [31]. In low-molecular-weight resins (Ep1), the high concentration of crosslinking nodes facilitates nucleophilic dehydration and chain scission reactions initiated by the amine nitrogen at relatively low temperatures (300–350 °C). Conversely, in high-molecular-weight resins (Ep4 and Ep5), the lower crosslink density (higher M_n_) and the predominance of long DGEBA oligomeric segments favor a shift in the degradation mechanism toward the homolytic scission of glyceridyl and ether bonds at higher temperatures [31]. The presence of long crosslink distances and a high content of hydroxyl groups within the polymer network structure facilitates polymer degradation and initiates a stepwise thermo-oxidative decomposition of macromolecular chain segments that differ in their thermal stability.

### 3.2. Characterization of Intumescent Coatings Based on APP/PER/MEL IS

**Figure 5:**
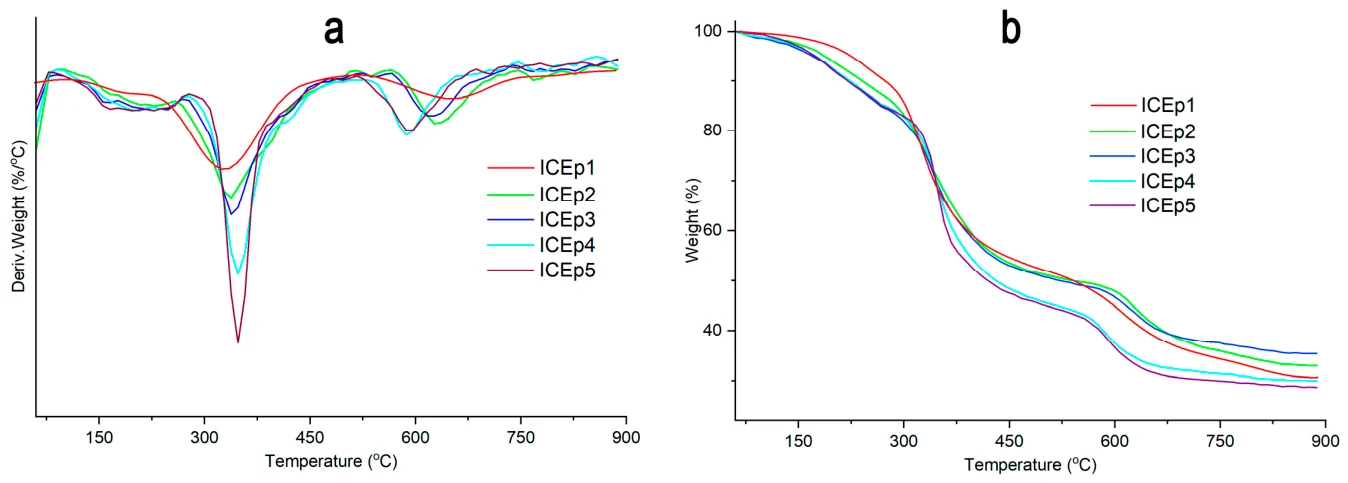
DTG (**a**) and TG-curves (**b**) of the intumescent coatings included APP/PER/MEL IS under O_2_ atmosphere.

Intumescent coatings were formulated using the investigated epoxy resins as binders, employing APP/PER/MEL IS. TGA of the resulting intumescent coatings (ICEp1–ICEp5) revealed significant differences in their thermal degradation behavior, which were strongly dependent on the type of epoxy resin employed. All the studied specimens exhibited a typical three-stage mass loss pattern characteristic of intumescent materials (Figure 5 and Table 8).

The first stage of mass loss, up to 300 °C, is attributed to the initial dehydration of the flame retardants and the incipient decomposition of the polymer matrix. At this stage, a clear trend is observed: as M_n_ of the resin increases, the onset temperature of 5% mass loss (T_5%_) of the coating decreases, while the temperature of the maximum degradation rate (T_max1_) shifts to higher values. This shift indicates a change in the dominant degradation mechanism. For low-molecular-weight resins, degradation is initiated by nucleophilic reactions involving the nitrogen atoms of the curing agent, whereas for high-molecular-weight resins, the cleavage of glyceridyl and ether bonds predominates at higher temperatures [32].

During the second stage, spanning the 300–500 °C, range, active decomposition of the polymer matrix occurs concurrently with the formation of a char layer. In this region, the sample based on Ep1 exhibits the lowest rate of mass loss, which is ascribed to its high degree of crosslinking and the highest density of the polymer network. This structural feature facilitates more effective retention of carbonaceous material during the early stages of pyrolysis. As the molecular weight of the binder increases, the rate of mass loss rises accordingly.

At the final stage, above 500 °C, the degradation of the previously formed char layer takes place. During this phase, ICEp1 sample undergoes the most rapid mass loss, as evidenced by a broad and diffuse peak on the DTG curve. The expanded char layers derived from the high-molecular-weight resins Ep4 and Ep5 likewise exhibit limited thermo-oxidative stability, as they reach their maximum rate of mass loss (T_max2_) at lower temperatures during this stage. The low char yield and limited stability of the residual char layer for these samples are attributed to the substantial mass loss occurring during the second degradation stage. This behavior is associated with the high content of uncrosslinked oligomeric fractions or the presence of long oligomeric segments within the network, which undergo rapid depolymerization without contributing to the stabilization of the char structure. Furthermore, the increased content of secondary hydroxyl groups in the oligomer chains serves as initiation centers for dehydration reactions catalyzed by the amine nitrogen. The reduction in crosslink density (increase in M_n_) enhances segmental mobility, facilitating nucleophilic cleavage reactions and radical rearrangements, thereby accelerating the overall degradation process.

Consequently, the medium-molecular-weight resins (ICEp2 and ICEp3 samples) prove to be the most suitable binders in terms of forming a thermally stable char residue, thereby providing superior barrier protection under high-temperature exposure.

To evaluate the chemical composition of the expanded char layers formed after cothermal exposure, FTIR spectroscopy was performed on the intumescent char residues obtained from the coatings based on the five epoxy resins (Figure S6 and Table S1). The FTIR spectra of all samples exhibited nearly identical absorption profiles, indicating that the molecular weight of the epoxy binder does not significantly influence the chemical structure of the resulting intumescent char. the intumescent char is predominantly composed of inorganic phosphate and borate species formed from the flame retardants, while the contribution of aromatic structures from the epoxy resin remains minor. The low intensity of aliphatic C–H and aromatic C=C bands indicates that the polymer matrix largely decomposes and volatilizes during the intumescence process, while the phosphorus-containing species actively crosslink the residual carbonaceous material, forming a thermally stable P–O–C network.

**Figure 6:**
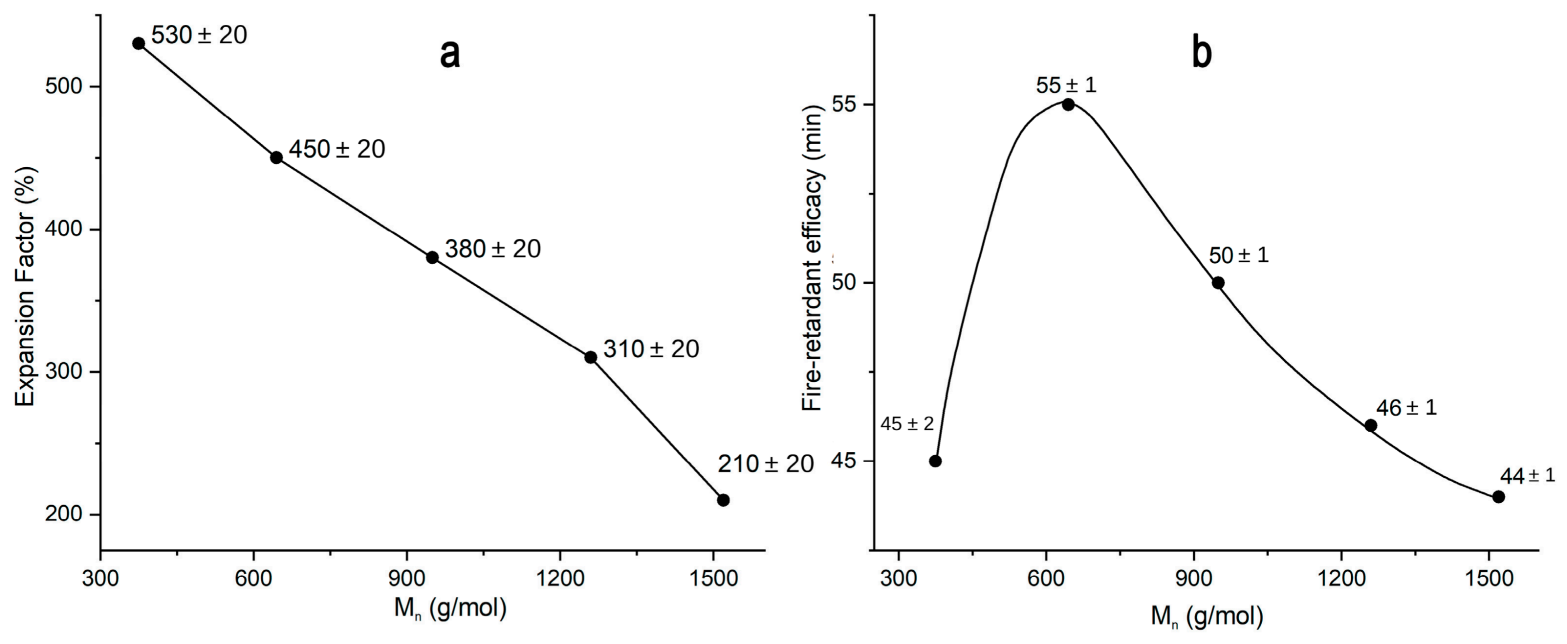
The influence of the molecular weight of the epoxy binder on the expansion factor (**a**) and fire-retardant efficacy (**b**) of the intumescent coatings included APP/PER/MEL intumescent system.

**Figure 7:**
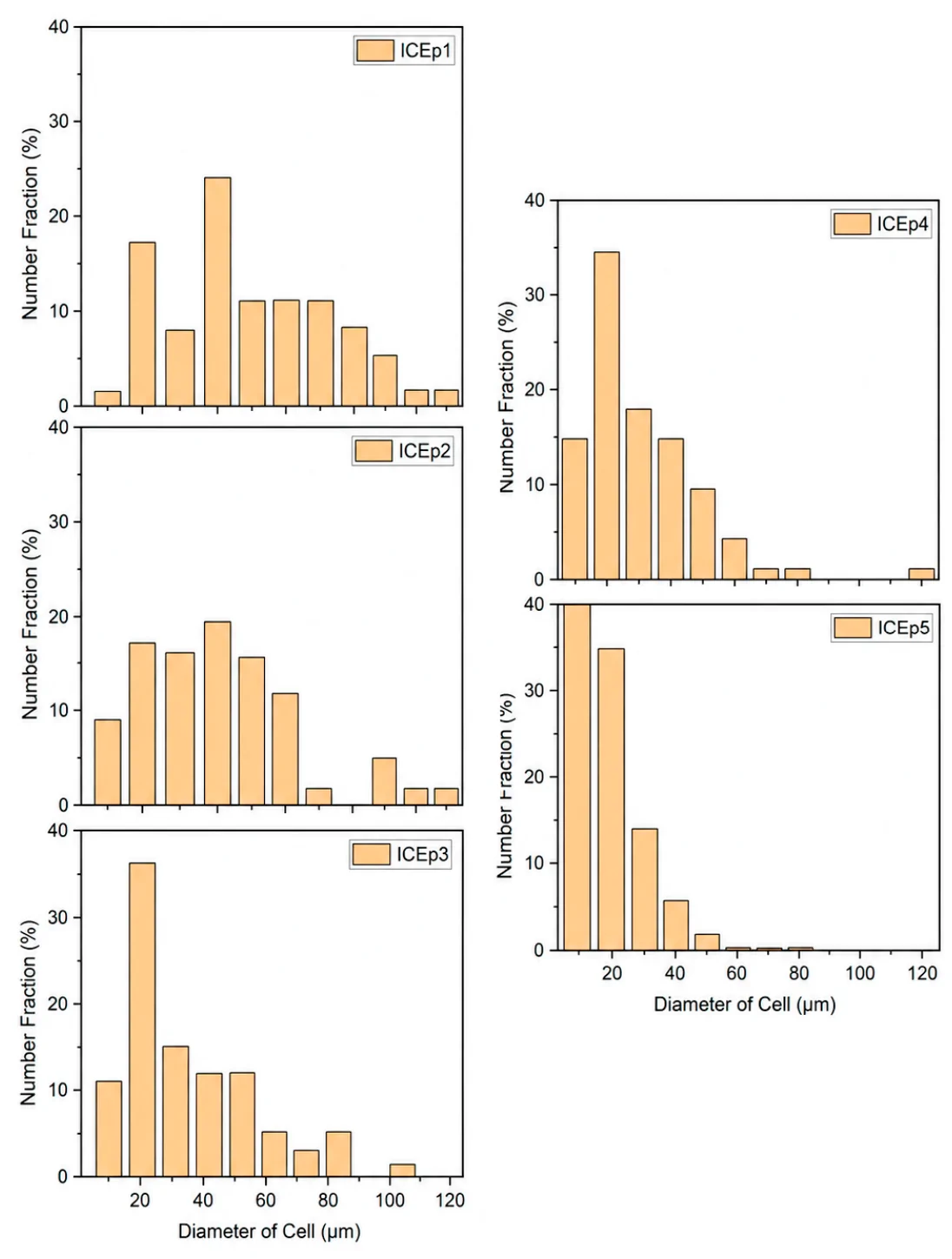
Cell diameter distributions in the char layers of APP/PER/MEL-based intumescent coatings formulated with epoxy resins Ep1–Ep5 of different molecular weights.

**Figure 8:**
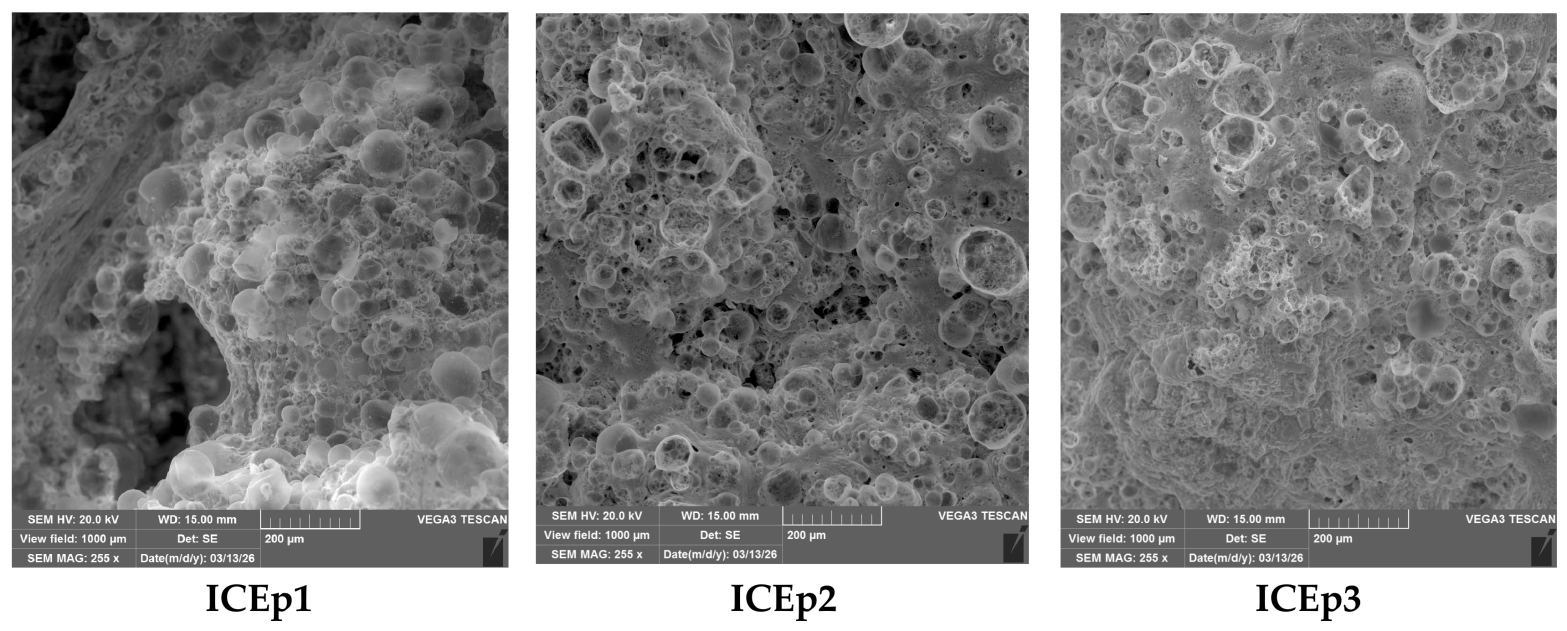

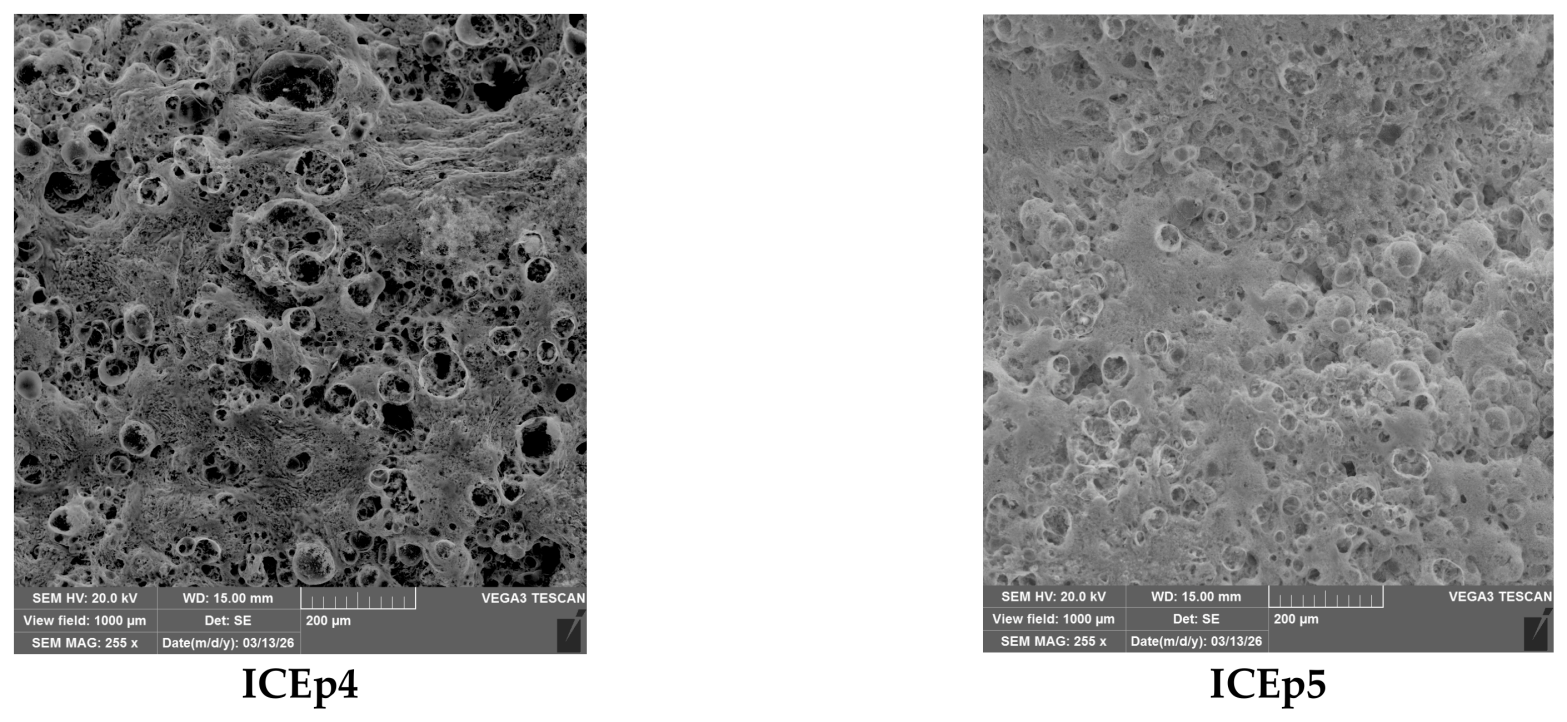
SEM micrographs of the char layer morphology obtained after combustion at 600 °C for the coatings based on the investigated epoxy resins.

Based on the fire test results presented in Figure 6, it was established that the expansion factor of the coating decreases with increasing M_n_ of the binder. Quantitative analysis of the pore structure further confirmed a systematic change in the cellular morphology of the expanded char. The pore-size distributions presented in Figure 7 show a progressive shift toward smaller pores with increasing molecular weight of the epoxy binder, while the corresponding mean equivalent pore diameters are summarized in Table 9. Thus, the reduction in the expansion factor is accompanied by a decrease in the characteristic pore size of the expanded char. This behavior is attributed to the rise in melt viscosity with increasing content of aromatic fragments within the polymer network structure. Such an increase in viscosity restricts the diffusion of evolving gases through the condensed phase, thereby inhibiting the normal foaming of the carbonized mass. The SEM micrographs presented in Figure 8 provide complementary information on the surface morphology of the expanded char and demonstrate that, with increasing M_n_, the char surface becomes more compact, with a greater proportion of continuous and monolithic regions. This indicates a direct correlation between pore size and expansion factor; however, the relationship between the cellular structure of the char and fire-retardant efficacy is non-linear and follows a characteristic optimum. An intermediate pore-size distribution and a moderate expansion factor provide the most favorable balance between thermal insulation and structural integrity, whereas excessive expansion and the formation of large cavities lead to structural defects that impair the barrier properties of the char.

The optimal fire-retardant efficacy, corresponding to a fire resistance time of 55 min, was achieved for the coating based on the medium-molecular-weight resin Ep2 (*Mn* = 645 g/mol) at moderate values of the expansion factor. The pore-size distribution and mean equivalent pore diameter *d_eq_* 41 µm obtained for this sample indicate the formation of a comparatively balanced cellular structure, which is consistent with its superior fire-retardant performance. In contrast, the use of low-molecular-weight Ep1 (*M*n = 375 g/mol) and high-molecular-weight Ep4 and Ep5 resins (*Mn* = 1260 and 1520 g/mol, respectively) as binders results in the formation of distinct types of structural defects within the expanded char layer. In the former case, the excessively low melt viscosity of the system, combined with the rapid and intense gas evolution during the early stages of pyrolysis, promotes the formation of macrocavities. Although the low-molecular-weight resin yields the highest expansion factor, its pore-size distribution is shifted toward larger pore diameters *d*_eq_ = 51 μm and contains a substantial contribution from large cells, consistent with the formation of macroscale cavities within the expanded structure. These large-scale voids compromise the mechanical integrity of the expanded char and lead to its premature collapse under thermal or mechanical stress.

**Figure 9:**
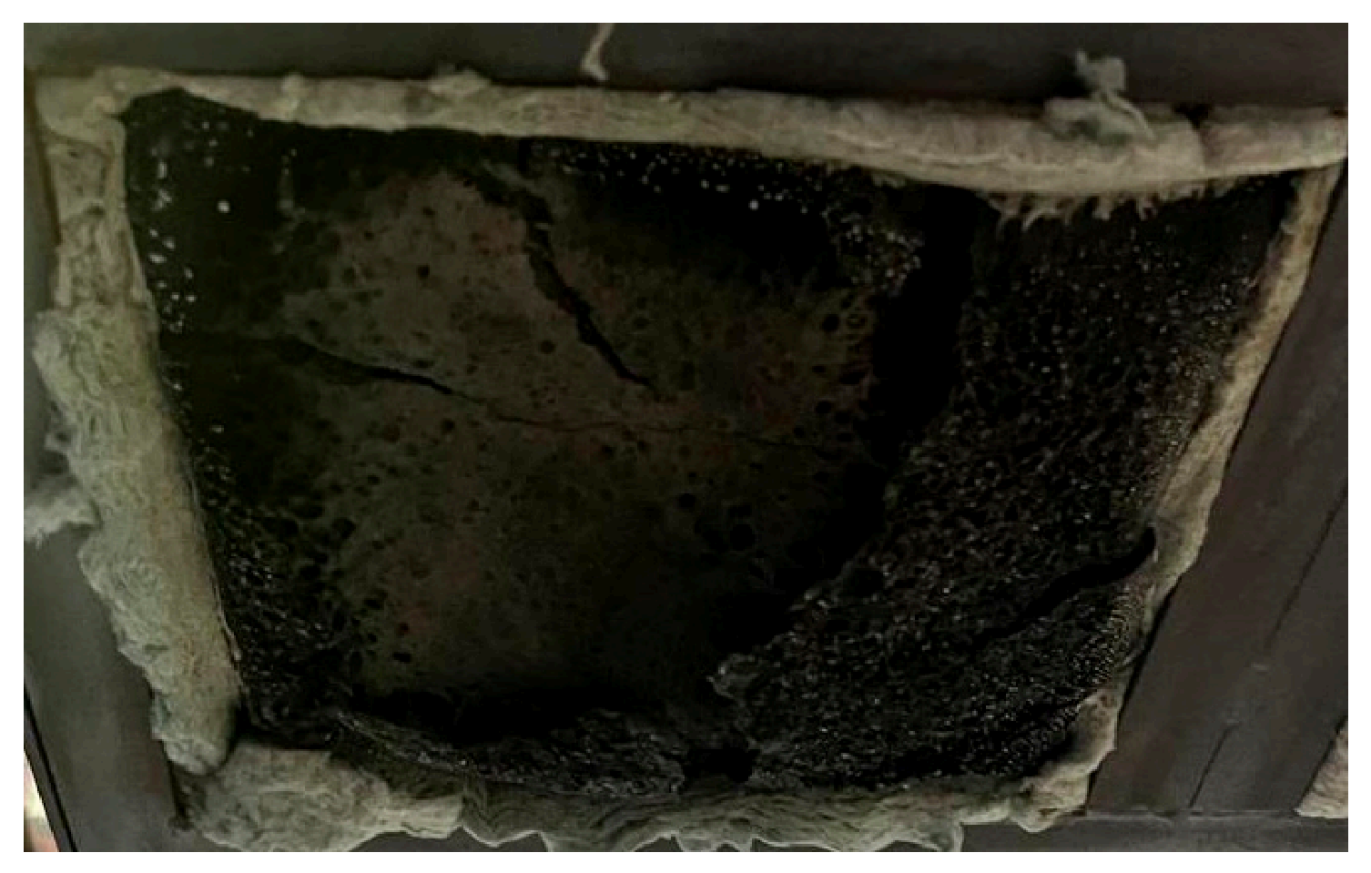
Visual appearance of the coating specimen ICEp5 following the fire-retardant efficacy tests.

At the opposite end of the molecular-weight range, Ep4 and Ep5 form chars characterized by smaller pore sizes (*deq* = 25 and 17 μm, respectively) and a more compact surface morphology; however, excessive melt viscosity suppresses uniform expansion and promotes the development of through-thickness cracks. These cracks extend down to the substrate, thereby providing a direct pathway for heat and mass transfer, which adversely affects the overall fire-retardant efficacy of the coating (Figure 9).

The established trends are in good agreement with the previously obtained data for intumescent coatings based on PVA as a binder [20]. Accordingly, general trends in the influence of binder molecular weight on the fire-retardant properties of coatings within the APP/PER/MEL IS can be inferred.

### 3.3. Characterization of Intumescent Coatings Based on EG

The mechanism of expanded char formation in intumescent coatings based on EG differs fundamentally from that of conventional systems relying on the classic APP/PER/MEL intumescent triad. In EG-based systems, volume expansion occurs primarily through the structural transformation of the filler itself upon thermal exposure, rather than through the gas-induced foaming of a carbonized polymer matrix. The expansion of EG is driven by the rapid release of intercalated compounds from the layered graphite structure, which causes the graphite flakes to exfoliate and expand significantly along the c-axis [33,34]. Consequently, the polymer matrix in such coatings acts primarily as a binder that holds the expanded flakes together, rather than as an active participant in the chemical reactions that form the char network [35].

To investigate the influence of the binder molecular weight on the fire-retardant properties of such EG-containing coatings, intumescent formulations were prepared using the investigated epoxy resins with a fixed EG loading of 15 wt.% (ICEp1/EG–ICEp1/EG).

The FTIR spectra (Figure S7 and Table S2) of the EG-based intumescent chars confirm that the organic polymer matrix (epoxy resin) plays no active role in the construction of the carbonaceous framework. The virtual absence of aliphatic C–H and aromatic C=C bands indicates that the binder completely decomposes and volatilizes during the intumescence process. The char is formed almost exclusively by expanded graphite flakes, with minor contributions from inorganic phosphate (from APP) and borate (from zinc borate) species.

**Figure 10:**
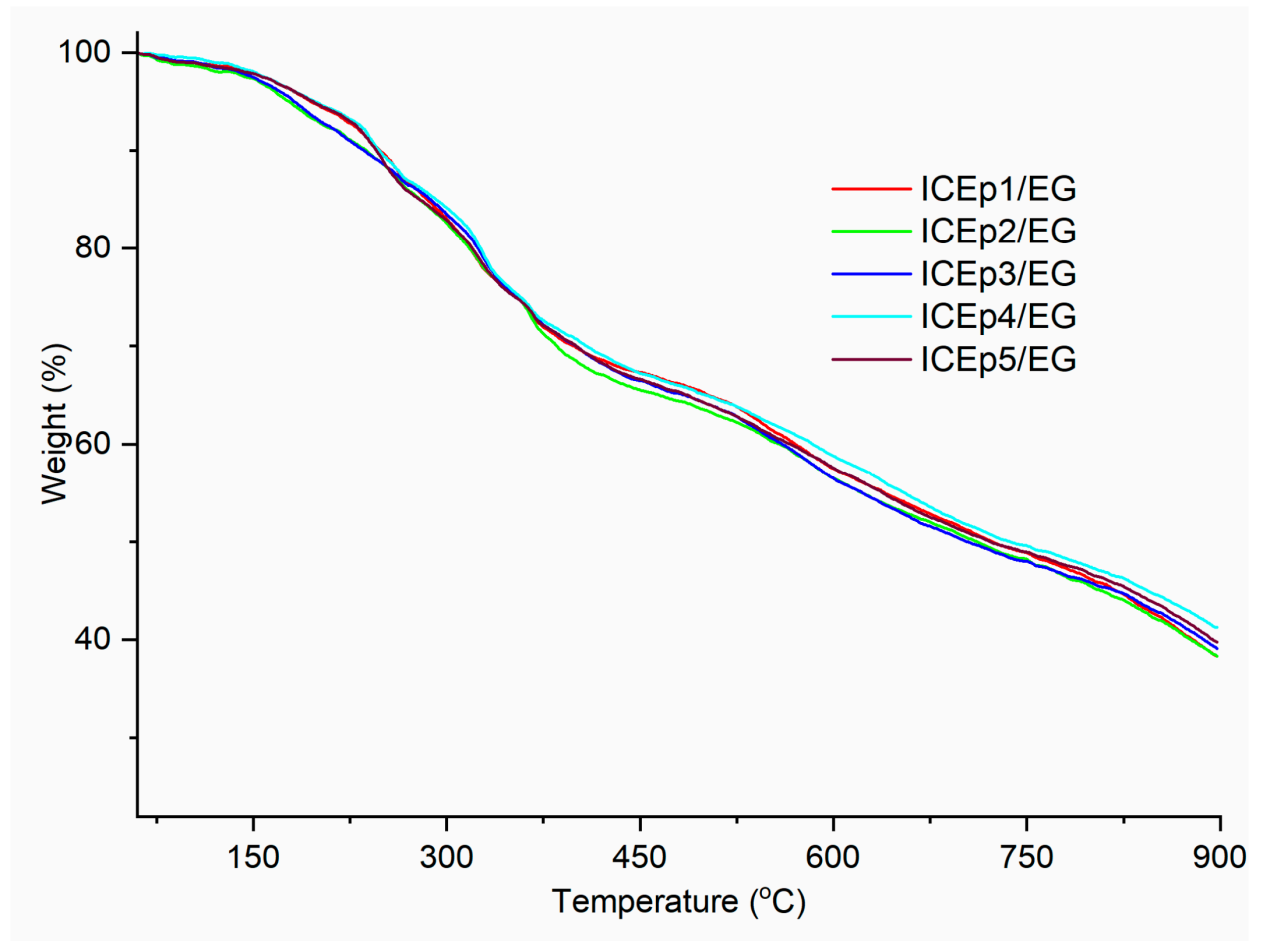
TG-curves of the EG-based intumescent coatings under O_2_ atmosphere.

The TGA results indicated that the variation in Mn did not significantly alter the overall degradation profiles of the coatings; the TGA curves for all samples remained closely grouped and exhibited similar mass loss trends (Figure 10). Notably, this observation contrasts with the distinct degradation behavior observed for the APP/PER/MEL-based coatings (Figure 5), where the molecular weight of the binder strongly influenced the decomposition pattern. The presence of EG effectively levels out these differences in the degradation character of the compounds, making the thermal decomposition of the composite largely independent of the binder molecular weight. This can be explained by the fact that the thermal degradation of the epoxy matrix is largely masked by the early-stage exfoliation of EG, which occurs rapidly and dominates the mass loss events in the 250–350 °C range. Furthermore, the carbonaceous residue formed from the polymer matrix contributes negligibly to the overall char yield in EG-based systems, as the char structure is predominantly built from the expanded graphite flakes.

**Figure 11:**
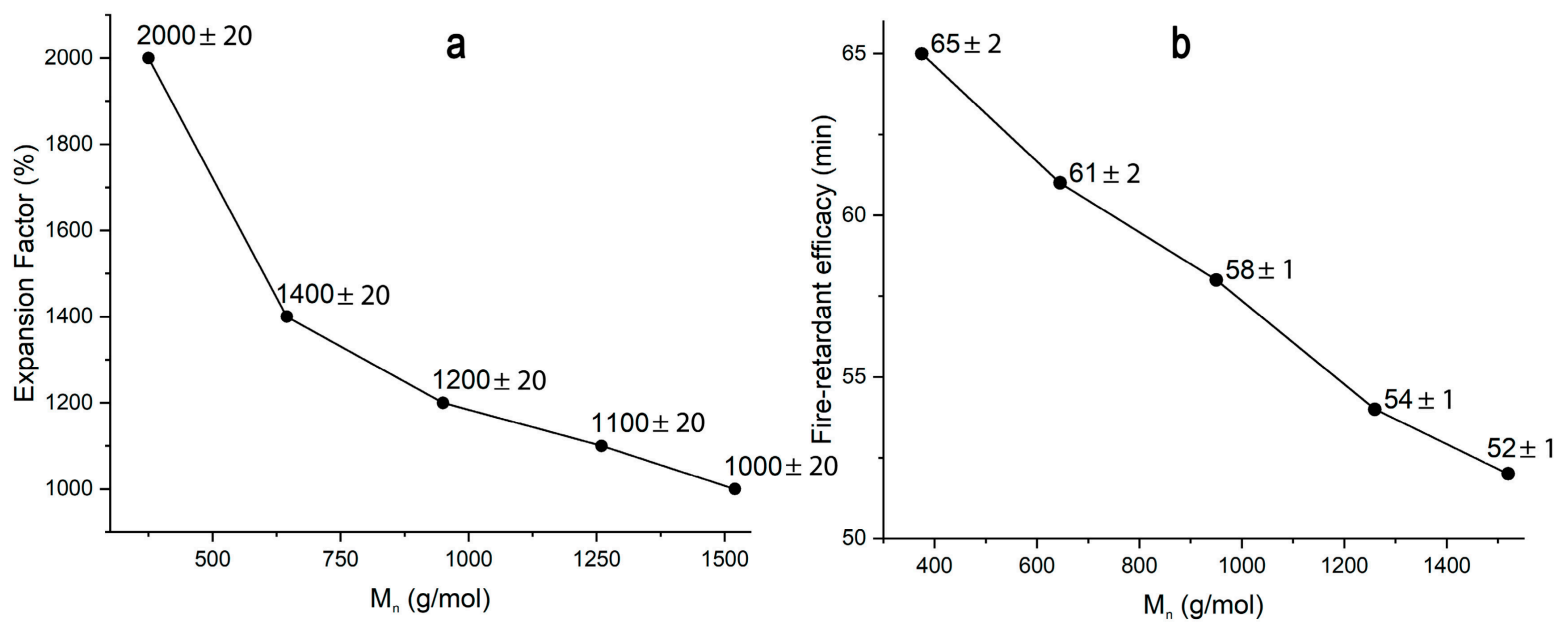
The influence of the molecular weight of the epoxy binder on the expansion factor (**a**) and fire-retardant efficacy (**b**) of the EG-based coatings.

**Figure 12:**
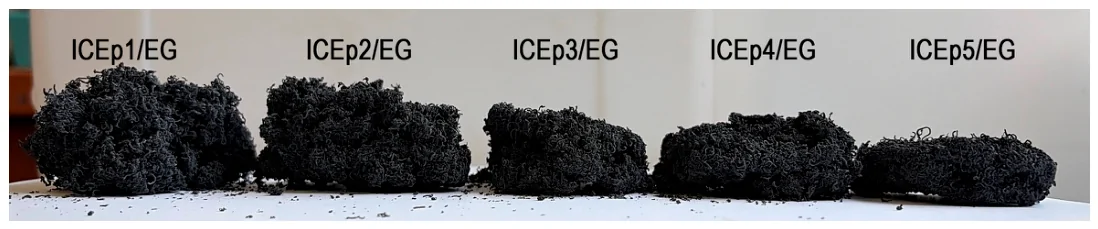
Visual appearance of the EG-based coating specimens obtained after calcination at 600 °C.

However, the fire test results revealed a pronounced inverse correlation between the molecular weight of the polymer matrix and both the expansion factor and the fire-retardant efficacy of the coatings (Figure 11 and Figure 12). As clearly demonstrated by the experimental data, the expansion factor decreased from nearly 2000% for the lowest-molecular-weight resin (Ep1) to approximately 1000% for the highest-molecular-weight resin (Ep5). Correspondingly, the fire-retardant efficacy showed a consistent decline, from 65 min for Ep1 to approximately 52 min for Ep 5. The most effective fire-retardant performance was achieved with the lowest-molecular-weight resin, Ep1.

This behavior is attributed primarily to the increased melt viscosity of the polymer matrix with rising M_n_. As Mn of the epoxy resin increases, the proportion of aromatic fragments within the polymer backbone increases. The chemical structure of the DGEBA oligomer (Figure 2) contains a bisphenol A unit with two aromatic rings; as chain length increases, the total content of aromatic fragments per unit mass grows. Consequently, the relative content of aliphatic fragments originating from the TETA hardener decreases, since the amount of hardener required for stoichiometric curing is reduced (Table 1). Thus, with increasing Mn, the polymer matrix contains a higher fraction of heat-stable aromatic structures and a lower fraction of thermally labile aliphatic segments. The increased aromatic content also affects the structure of the carbonaceous residue formed during thermal decomposition. Aromatic structures favor the formation of thermally stable carbonaceous residues and can promote the development of a more cohesive carbon-rich framework. However, in the EG-based system, where the protective char is formed predominantly by the physical expansion and stacking of graphite flakes, the contribution of the epoxy-derived carbonaceous residue to the overall char structure is limited. Therefore, the higher aromatic content does not compensate for the rheological constraint imposed by the more viscous polymer matrix. As a result, after the initial decomposition of the aliphatic fragments of TETA, the remaining melt becomes increasingly viscous due to the predominance of rigid aromatic units. This high viscosity restricts the unhindered expansion and rearrangement of the EG particles, resulting in a denser and less expanded protective layer, while the expansion of the oxidized graphite particles within this more rigid and compact carbonaceous matrix can disrupt its continuity, generating local defects and discontinuities in the protective char. Consequently, the progressively increasing rigidity of the matrix at higher M_n_ not only suppresses EG expansion but may also compromise the structural integrity and continuity of the resulting protective layer, thereby contributing to the observed decrease in fire-retardant efficacy.

Moreover, the thermal degradation products of the epoxy binder do not actively participate in the construction of the carbonaceous framework in EG-based systems. In contrast to APP/PER/MEL coatings, where the polymer matrix contributes to the formation of a continuous, chemically bonded char network, the char structure in EG-based coatings is formed almost exclusively by the expanded graphite flakes. The role of the epoxy binder is limited to providing a cohesive medium that holds the expanded flakes together, rather than contributing to the chemical composition or thermal stability of the char itself. Therefore, a lower-molecular-weight resin with lower melt viscosity not only facilitates greater expansion but also ensures better distribution and cohesion of the EG particles, leading to a more structurally uniform and effective barrier.

In summary, unlike the APP/PER/MEL system, where an intermediate molecular weight provides the optimal balance between foaming and structural integrity, EG-based coatings benefit from the lowest molecular weight resins, which minimize resistance to EG expansion and maximize the final char volume.

## 4. Conclusions

This study systematically investigated the effect of the molecular weight of a bisphenol A-based epoxy binder on the fire-retardant performance of intumescent coatings based on two fundamentally different intumescent mechanisms: the classical APP/PER/MEL system and EG.

For the APP/PER/MEL-based coatings, the molecular weight of the epoxy resin exerted a dual influence on fire-retardant efficacy. The low-molecular-weight resin Ep1 (*M*n = 375 g/mol) promoted rapid and intense gas evolution, which, combined with the low melt viscosity, resulted in the formation of macrocavities, a high mean equivalent pore diameter (*d*_eq_ = 51 μm), and premature collapse of the char layer. In contrast, the high-molecular-weight resins Ep4 and Ep5 (*Mn* = 1260 and 1520 g/mol, respectively) generated a highly viscous melt that restricted uniform expansion and promoted through-thickness cracking of the char layer. These coatings exhibited a lower mean equivalent pore diameter (*deq* = 25 and 17 μm, respectively), indicating a less favorable pore structure and compromising the thermal-barrier function of the char. The highest fire-retardant efficacy, corresponding to 55 min of protection, was achieved with the medium-molecular-weight resin Ep2 (*Mn* = 645 g/mol), which provided a moderate expansion factor and a structurally intact char with an optimal pore structure (*d*_eq_ = 41 μm). These results demonstrate that the APP/PER/MEL system exhibits an optimum with respect to epoxy resin molecular weight.

In contrast, the EG-based coatings exhibited a monotonic inverse relationship between epoxy resin molecular weight and both the expansion factor and fire-retardant efficacy. The highest performance, corresponding to 65 min of protection, was obtained with the lowest-molecular-weight resin Ep1 (*M*n = 375 g/mol), which provided an expansion factor of approximately 2000%. Unlike the APP/PER/MEL system, in which the decomposition products of the binder contribute to the formation of the intumescent carbonaceous framework, the protective layer in the EG-based coatings is formed predominantly through the physical expansion and stacking of graphite flakes. Consequently, the contribution of the epoxy-derived carbonaceous residue to the overall char structure is limited, and the fire-retardant performance is governed primarily by the ability of the binder matrix to accommodate and sustain EG expansion.

From a practical formulation perspective, these findings indicate that the selection of bisphenol A-based epoxy binders for intumescent fire-protective materials for steel structures should be guided by the expansion characteristics of the intumescent system. For formulations capable of achieving high expansion ratios (>1200%), low-molecular-weight epoxy resins with a molecular weight of approximately 300–400 g/mol are recommended. For formulations characterized by moderate expansion ratios (400–600%), epoxy resins with intermediate molecular weights in the range of 400–700 g/mol are more appropriate.

## Figures and Tables

**Table 1:** Characteristics of epoxy resins.

Grade	Epoxy Resin	CAS №	M_n_ (g/mol) ^1^	Physical State	Hardener Content (phr)
NPEL-128	Ep1	25068-38-6	375	Liquid	12.8
NPSN-136X80	Ep2	25036-25-3	645	80% solution in xylene	7.5
NPSN-901X75	Ep3	25036-25-3	950	75% solution in xylene	5.1
NPES-902	Ep4	25036-25-3	1260	Solid	3.8
NPES-903	Ep5	25036-25-3	1520	Solid	3.1

^1^ The theoretical M_n_ was derived from the epoxy equivalent weight.

**Table 2:** Composition based on APP/PER/MEL.

Ingredients	Content (wt. %)
Epoxy resin (100% solids)	30.0
Ammonium polyphosphate	18.4
Pentaerythritol	5.0
Melamine	7.0
Zinc borate	18.0
Titanium dioxide	5.0
Xylene	10.0
Functional additives	7.0
∑	100.0

**Table 3:** Composition based on EG.

Ingredients	Content (wt. %)
Epoxy resin (100% solids)	25.5
Expandable graphite	15.0
Ammonium polyphosphate	18.0
Zinc borate	18.0
Magnesium silicate fiber	5.0
Antimony (III) oxide	1.5
Xylene	10.0
Functional additives	7.0
∑	100.0

**Table 4:** Oligomeric composition of the epoxy resins.

Degree of Oligomerization	Content (wt. %)				
	Ep1	Ep2	Ep3	Ep4	Ep5
*n* = 0 (DGEBA)	84.3	29.1	20.0	15.0	10.1
*n* = 1	10.4	5.3	4.1	4.0	3.0
*n* = 2	2.3	33.2	34.1	26.2	20.6
*n* = 3		18.4	10.3	10.1	11.2
*n* = 4		10.8	18.4	17.7	31.9
*n* = 5			7.2	7.2	8.4
*n* ≥ 6, impurities and non-elutable material	3.1	3.2	5.9	19.8	14.8

**Table 5:** Determination of the degree of oligomerization (*n*) of epoxy resins Ep1–Ep5 by NMR spectroscopy.

Grade	Ep1	Ep2	Ep3	Ep4	Ep5
*I* (CH_Ep_)/*I* (CH_Ar_)	2.4	4.0	5.4	7.3	9.3
*n*	0–1	1	1–2	2–3	≈4

Note. *I* (CH_Ep_)—the signal intensity of the methine proton of the epoxy ring at δ_H_ = 3.35–3.40 ppm; *I* (CH_Ar_)—the signal intensity of the methine protons of the aromatic ring at δ_H_ = 6.85–6.90 ppm; calculated values of *I* (CH_Ep_)/*I* (CH_Ar_): 2 for *n* = 0; 4 for *n* = 1; 6 for *n* = 2; 8 for *n* = 3; 10 for *n* = 4.

**Table 6:** Characteristic glass transition temperatures of the epoxy resins cured by TETA.

Sample	Ep1/TETA	Ep2/TETA	Ep3/TETA	Ep4/TETA	Ep5/TETA
T_eig_ (°C)	107	102	101	90	97
T_mg_ (°C)	113	106	105	95	101
T_efg_ (°C)	116	109	107	97	103

**Table 7:** TG and DTG data of the epoxy resins cured by TETA under O_2_ atmosphere.

Sample	Ep1/TETA	Ep2/TETA	Ep3/TETA	Ep4/TETA	Ep5/TETA
T_5%_ (°C)	308	175	140	162	138
T_max1_ (°C)	338	367	357		
T_max2_ (°C)		437	427	97	103
T_max3_ (°C)	547	558	558	557	561

**Table 8:** TG and DTG data of the intumescent coatings included APP/PER/MEL IS under O_2_ atmosphere.

Sample	ICEp1	ICEp2	ICEp3	ICEp4	ICEp5
T_5%_ (°C)	227	187	166	165	166
T_max1_ (°C)	328	338	338	348	347
T_max2_ (°C)	657	630	626	586	590
Char yield at 900 °C (%)	31	33	35	30	28

**Table 9:** Mean equivalent pore diameter of the expanded char samples.

Sample	ICEp1	ICEp2	ICEp3	ICEp4	ICEp5
*d_eq_* (µm)	51	41	34	25	17

## Data Availability

The original contributions presented in this study are included in the article/Supplementary Materials. Further inquiries can be directed to the corresponding author.

## Supplementary Materials

The following supporting information can be downloaded at https://www.mdpi.com/article/10.3390/polym18182201/s1, Figure S1. ^1^H NMR spectrum of NPEL-128 (Ep1); Figure S2. ^1^H NMR spectrum of NPSN-136X80 (Ep2); Figure S3. ^1^H NMR spectrum of NPSN-901X75 (Ep3); Figure S4. ^1^H NMR spectrum of NPES-902 (Ep4); Figure S5. ^1^H NMR spectrum of NPES-903 (Ep5); Figure S6. FTIR spectra of the intumescent char residues obtained from the APP/PER/MEL-based coatings after combustion at 600 °C; Figure S7. FTIR spectra of the intumescent char residues obtained from the EG-based coatings after combustion at 600 °C; Table S1. Assignment of characteristic FTIR absorption bands for the APP/PER/MEL intumescent char residues; Table S2. Assignment of characteristic FTIR absorption bands for the EG-based intumescent char residues.
