# Supplementary material for: Tailoring the Fire-Retardant Efficacy of Intumescent Coatings via Optimal Selection of Epoxy Binder Molecular Weight
Source: Polymers (Basel). 2026 Sep 9;18(18):2201. doi: 10.3390/polym18182201 (PMC13611362; doi:10.3390/polym18182201)
Supplement: Supplementary file 1 [file polymers-18-02201-s001.zip › polymers-4504648-supplementary.pdf]

Tailoring the Fire-Retardant Efficacy of Intumescent Coatings via Optimal Selection of Epoxy Binder Molecular Weight

Daria Kuznetsova, Nikolay Yashin, Vyacheslav Subbotin and Victor Avdeev

Department of Chemistry, Lomonosov Moscow State University, Moscow 119991, Russia; mallolo1111@gmail.com (D.K.); subbotin.v@ograx.ru (V.S.); avdeev@highp.chem.msu.ru (V.A.)
* Correspondence: yashin.ni.v@gmail.com (N.Y.)

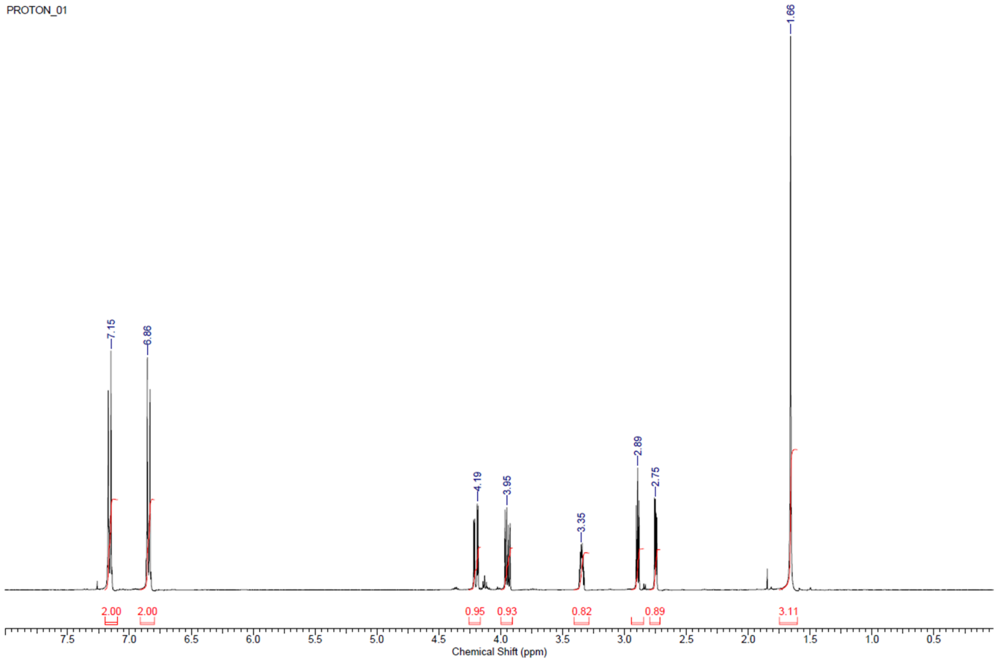

Figure S1. ^{1}H NMR spectrum of NPEL-128 (Ep1)

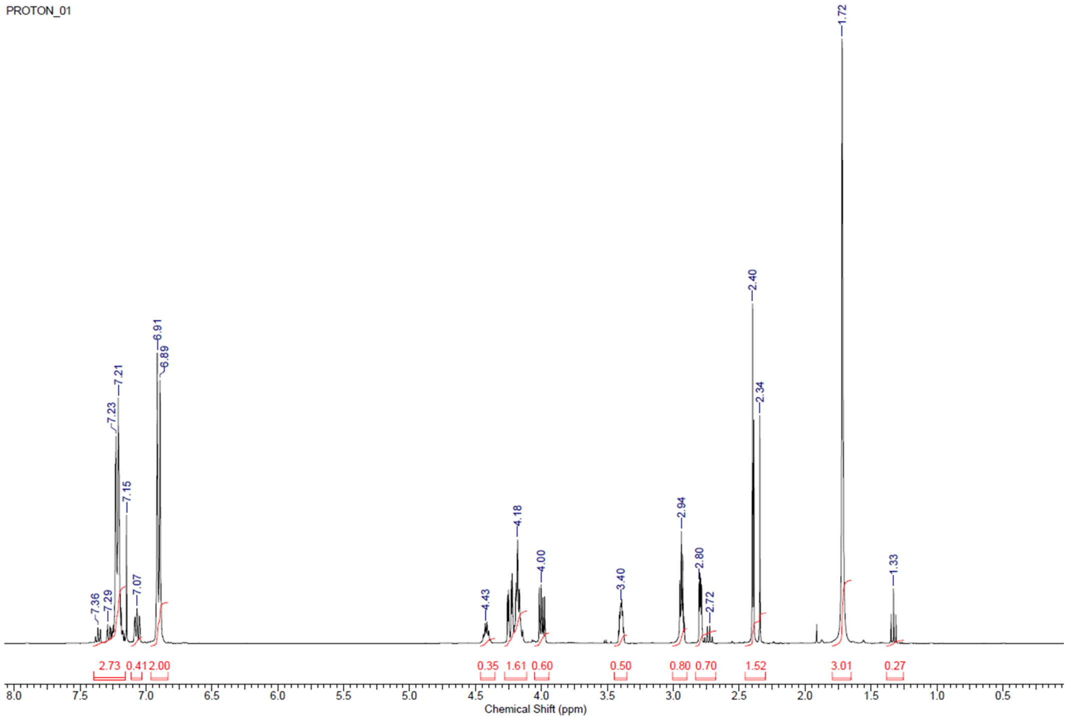

Figure S2. ^{1}H NMR spectrum of NPSN-136X80 (Ep2)

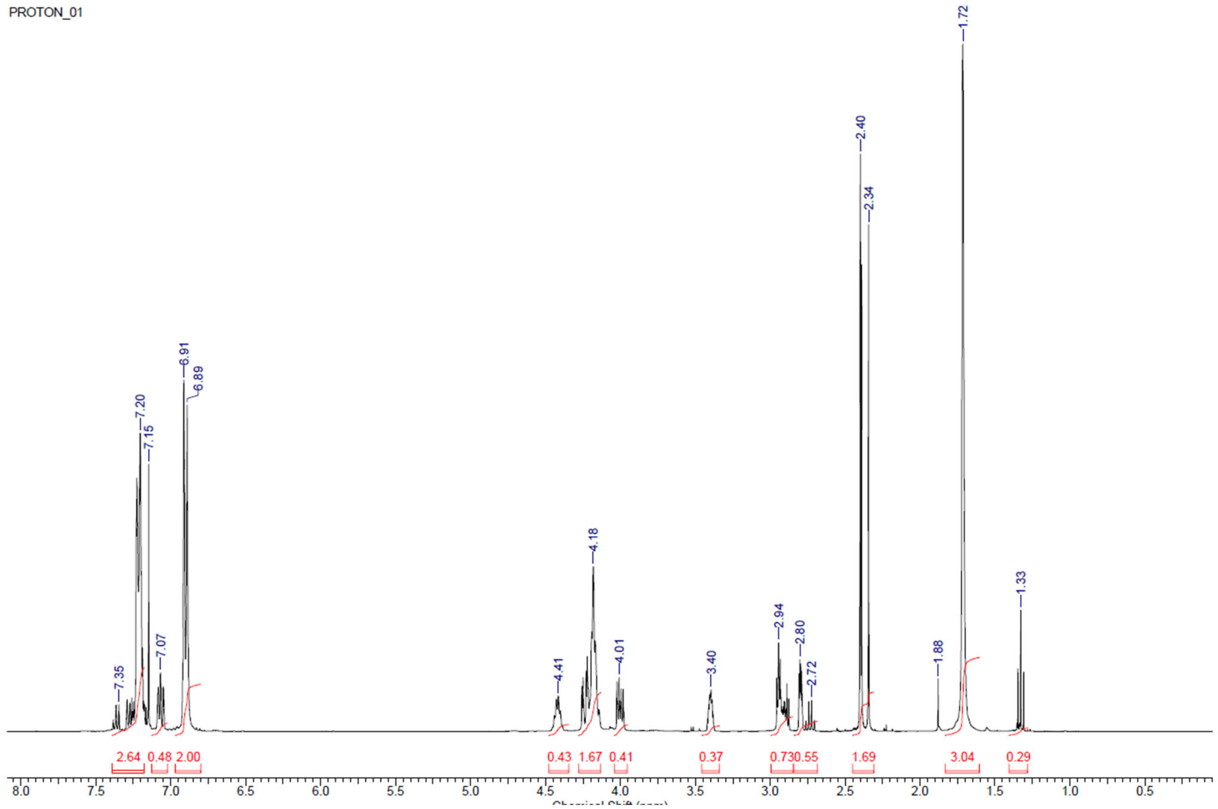

Figure S3. ^{1}H NMR spectrum of NPSN-901X75 (Ep3)

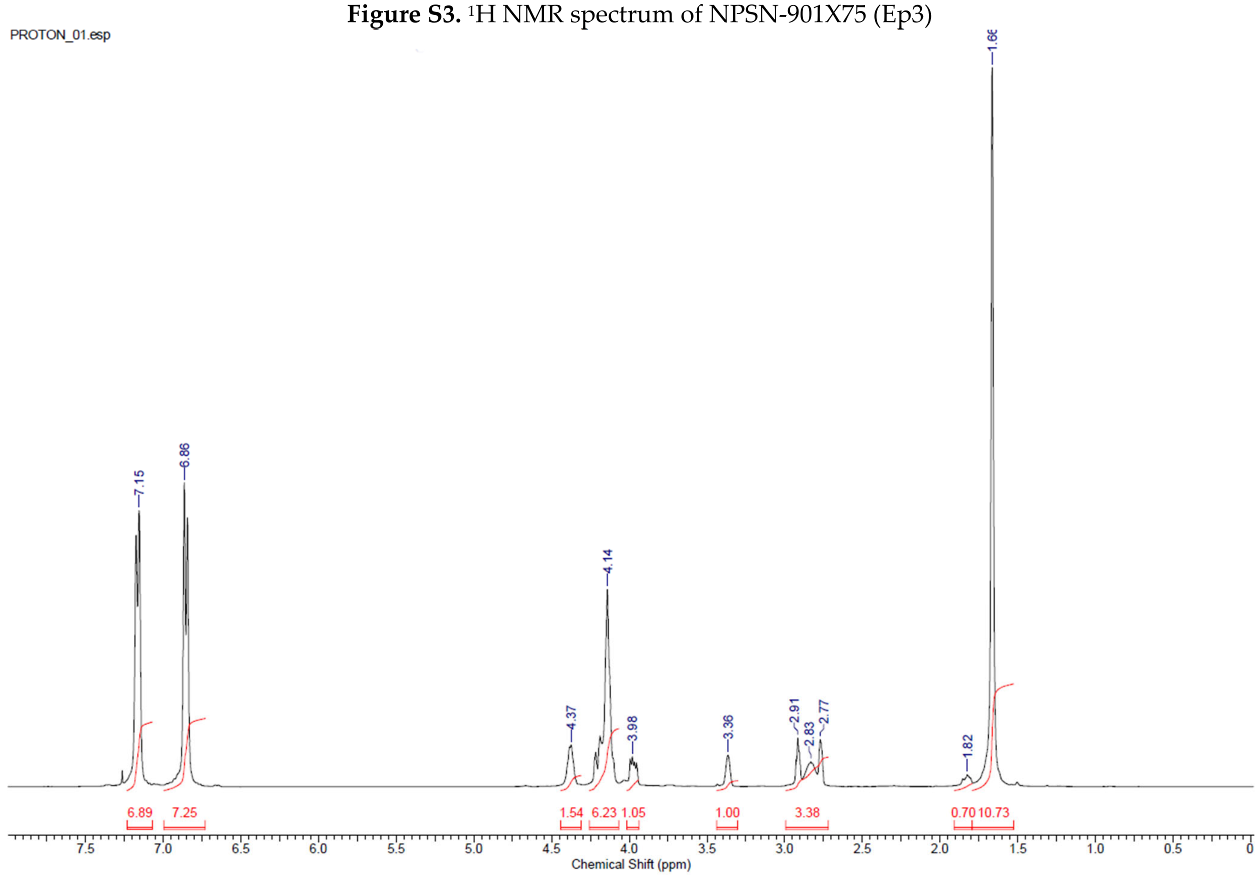

Figure S4. ^{1}H NMR spectrum of NPES-902 (Ep4)

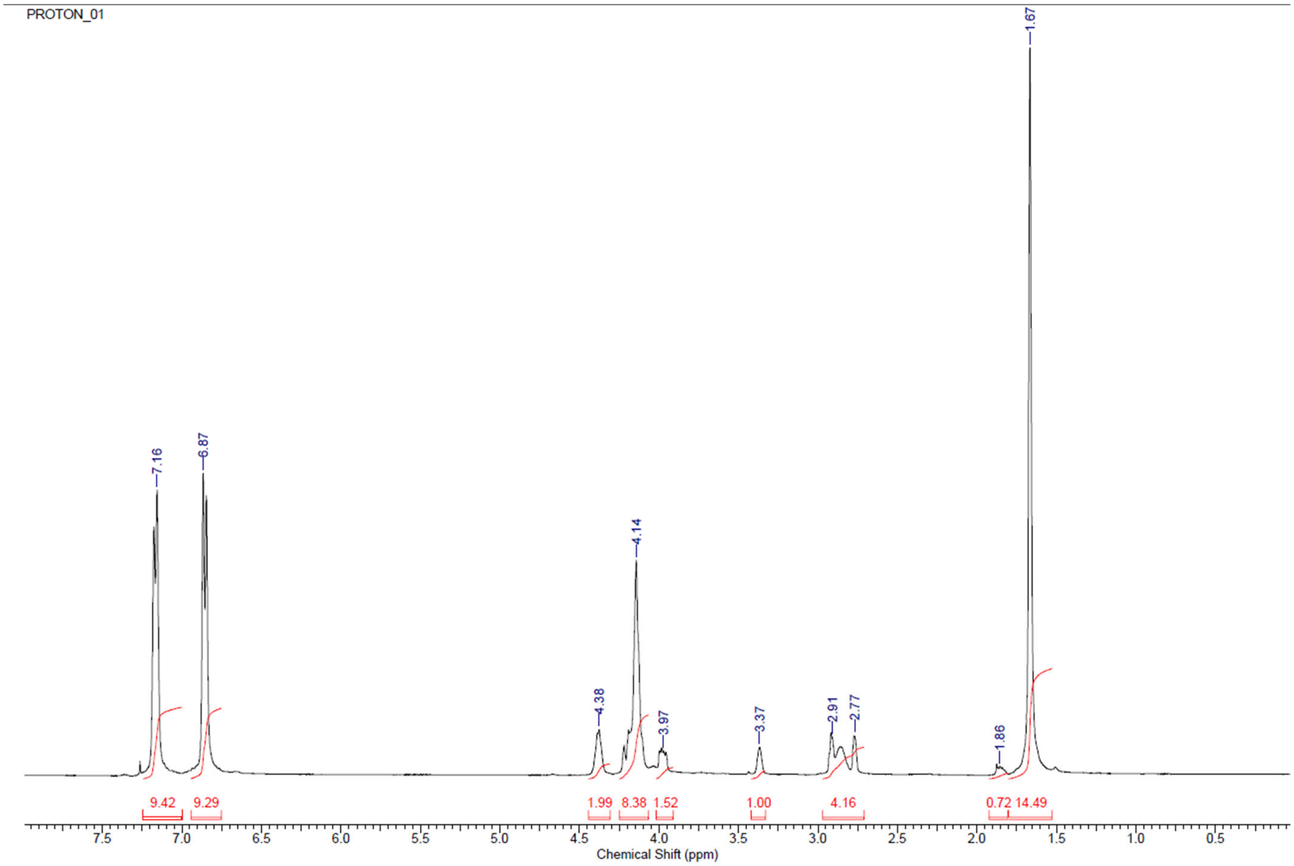

Figure S5. ^{1}H NMR spectrum of NPES-903 (Ep5)

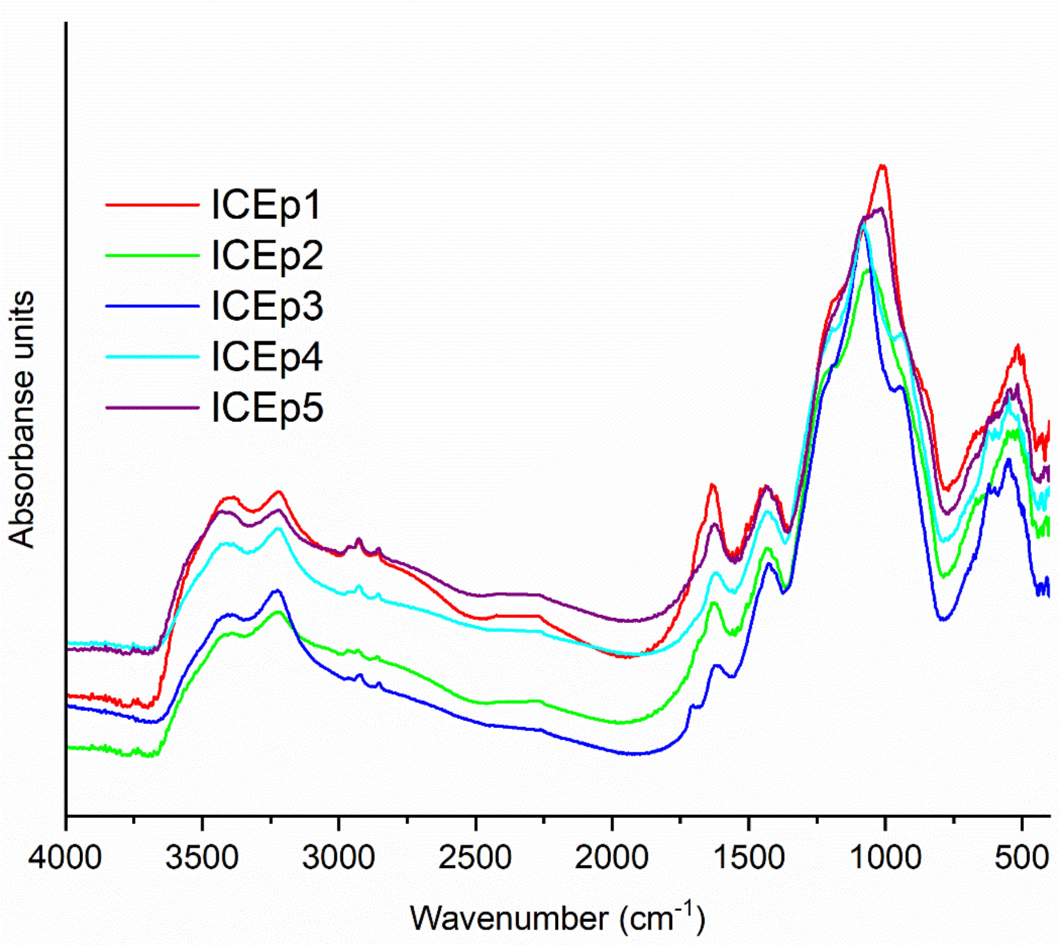

Figure S6. FTIR spectra of the intumescent char residues obtained from the APP/PER/MEL-based coatings after combustion at 600 °C.

Table S1. Assignment of characteristic FTIR absorption bands for the APP/PER/MEL intumescent char residues.

Peak position (cm^{-1})	Peak assigment
670–570	Aromatic C–H out-of-plane bending
800–700	Aromatic C–H out-of-plane bending / P–N / Ti–O
980–920 (shoulder)	P–OH bending / P–O–C (symmetric) / P–N stretching
1080–1050 (vertex of the strongest band)	P–O–P / P–O–C / C–O–C stretching
1250–1200 (broad shoulder)	P=O stretching / C–O (phenolic and ether)
1500–1300	N–H bending / C–N stretching / P–O (asymmetric)
1400–600 (broad, unstructured)	Borate glass phases ($ZnO·B_2O_3$)
1640–1620	Aromatic C=C stretching
3000–2800 (very weak)	Aliphatic C–H stretching
3600–3200 (broad complex band)	O–H and N–H stretching vibrations

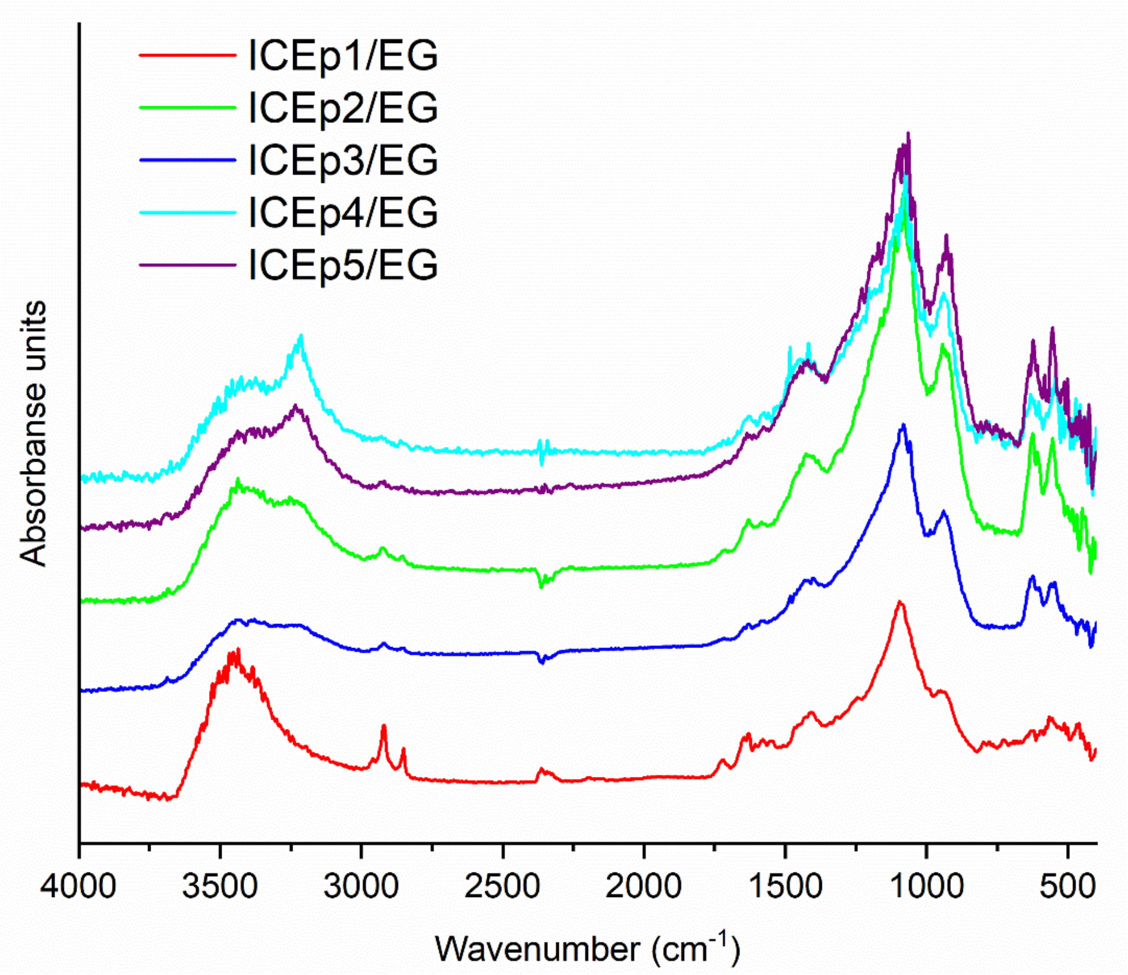

Figure S7. FTIR spectra of the intumescent char residues obtained from the EG-based coatings after combustion at 600 °C.

Table S2. Assignment of characteristic FTIR absorption bands for the EG-based intumescent char residues.

Peak position (cm^{-1})	Peak assigment
< 700 (sharp / structured)	Metal–O vibrations (Sb–O, Zn–O)
670–570 (weak)	Aromatic C–H out-of-plane bending / Sb–O
980–920 (shoulder)	P–OH bending / P–O–C (symmetric)
1080–1050 (vertex of the strongest band)	P–O–P / P–O–C stretching / C–O–C (ether)
1440–1420 (weak)	C–N / N–H bending
1600–1650 (very weak / absent)	Aromatic C=C stretching
1400–600 (broad, unstructured)	Borate glass phases ($ZnO{\cdot}B_2O_3$)
2850–2960 (very weak / absent)	Aliphatic C–H stretching
3400–3600 (broad, moderate)	Aliphatic C–H stretching
3600–3200 (broad complex band)	O–H stretching vibrations
